# LAG-3 as a Potent Target for Novel Anticancer Therapies of a Wide Range of Tumors

**DOI:** 10.3390/ijms23179958

**Published:** 2022-09-01

**Authors:** Natalia Sauer, Wojciech Szlasa, Laura Jonderko, Małgorzata Oślizło, Dominika Kunachowicz, Julita Kulbacka, Katarzyna Karłowicz-Bodalska

**Affiliations:** 1 Faculty of Pharmacy, Wroclaw Medical University, 50-556 Wroclaw, Poland; 2 Faculty of Medicine, Wroclaw Medical University, 50-367 Wroclaw, Poland; 3 Department of Molecular and Cellular Biology, Faculty of Pharmacy, Wroclaw Medical University, 50-556 Wroclaw, Poland; 4 Department of Drugs Form Technology, Faculty of Pharmacy, Wroclaw Medical University, 50-556 Wroclaw, Poland

**Keywords:** LAG-3, novel anticancer therapies, various tumor types

## Abstract

LAG-3 (Lymphocyte activation gene 3) protein is a checkpoint receptor that interacts with LSEC-tin, Galectin-3 and FGL1. This interaction leads to reduced production of IL-2 and IFN-γ. LAG-3 is widely expressed in different tumor types and modulates the tumor microenvironment through immunosuppressive effects. Differential expression in various tumor types influences patient prognosis, which is often associated with coexpression with immune checkpoint inhibitors, such as TIM-3, PD-1 and CTLA-4. Here, we discuss expression profiles in different tumor types. To date, many clinical trials have been conducted using LAG-3 inhibitors, which can be divided into anti-LAG-3 monoclonal antibodies, anti-LAG-3 bispecifics and soluble LAG-3-Ig fusion proteins. LAG-3 inhibitors supress T-cell proliferation and activation by disallowing for the interaction between LAG-3 to MHC-II. The process enhances anti-tumor immune response. In this paper, we will review the current state of knowledge on the structure, function and expression of LAG-3 in various types of cancer, as well as its correlation with overall prognosis, involvement in cell-based therapies and experimental medicine. We will consider the role of compounds targeting LAG-3 in clinical trials both as monotherapy and in combination, which will provide data relating to the efficacy and safety of proposed drug candidates.

## 1. Introduction

Lymphocyte-activation gene 3 (LAG-3/CD223) is a 503 amino acid protein, localized in the cell membrane [1]. The extracellular part of the molecule consists of four immunoglobulin-like domains (D1-D4) The protein is encoded by the *LAG3* gene [2]. Its expression is regulated by IL-2, IL-7 and IL-12A/B on T cells [3]. Interestingly, the 8-exon-gene lies adjacent to the CD4 gene on the 12 chromosome (12: 6.77–6.78). The structural similarity of both genes’ is about 20% [4]. The protein biosynthesis product undergoes several modifications. These include the cleavage by ADAM10 and ADAM17 metalloproteinases, leading to the secretion of Secreted LAG-3 (sLAG-3). ADAM10 catalyzes constitutive cleavage induced by T cell activation [4]. However, ADAM17 activity enhances by T cell receptor (TCR) signaling in a PRKCQ-dependent manner. Besides, the protein is glycosylated on N250, N188, C256 and N343 amino acids. 

The antigen is an immune checkpoint receptor, which regulates T cell functions. Expressed on activated T cells, the antigen acts similarly to programmed death receptor-1 (PD-1) or cytotoxic T lymphocyte antigen-4 (CTLA-4) in the inhibition of cytotoxic cell function [5]. 

**Figure 1:**
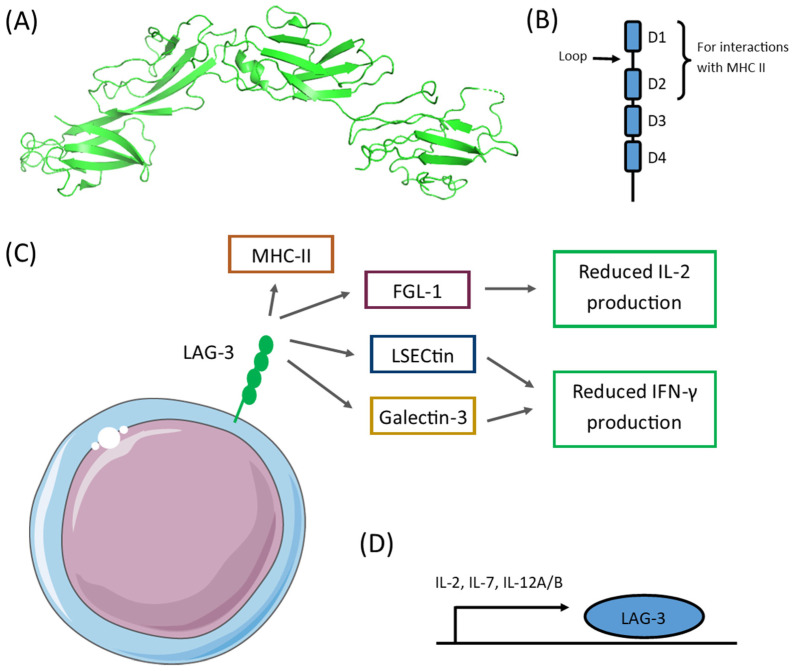
(**A**) Molecular structure of LAG-3 D1-D4 domains derived from PDB 7TZG; (**B**) LAG-3 membrane protein is composed of D1-D4 Ig domains, D1, D2 and loop are required for the interactions with MHC-II; (**C**) Lymphocytes interactions via LAG-3 with MHC-II, FGL-1, LSECtin, Galectin-3, which leads to the reduced production of IL-2 and IFN-γ; (**D**) Expression of LAG-3 regulated by IL-2, IL-7 and IL-12A/B.

LAG-3 remains a ligand for the major histocompatibility complex (MHC) class II. Via the interaction, LAG-3 inhibits cellular proliferation and T cell activation [6]. The protein also binds to fibrinogen-like protein 1 (FGL1) in the MHC-II independent pathway, which also delivers an inhibitory signal. Besides, 3A9 cells expressing LAG-3 reduced IL-2 secretion upon treatment with FGL1 [7]. The other ligands for LAG-3 are LSECtin and Galectin-3 derived from tumor and tumor stromal cells, respectively. Both ligands lead to the reduction of IFN-gamma production in LAG-3 sufficient cells [8,9]. LAG-3 blockade has demonstrated the ability to enhance the efficacy of PD-1 blockade [10]. Figure 1 summarizes the molecular structure of LAG-3, as well as its interactions and regulations of LAG-3 expression.

## 2. LAG-3 Expression

The lymphocyte activation gene 3 (LAG-3 or CD223) is expressed on Natural Killer (NK) cells, invariant NK T cells, Treg cells, and both CD4+ and CD8+ subsets of T lymphocytes upon activation by antigen [11].

It was found that LAG-3 cell surface expression is tightly regulated by extracellular cleavage in order to regulate its potent inhibitory activity [12]. 

The intracellular storage of LAG-3 and its close association with the microtubule organization center and recycling endosomes may facilitate its rapid translocation to the cell surface during T cell activation and help mitigate T cell activation [12]. LAG-3 is colocalized with CD4 in recycling endosomes, secretory lysosomes, and microtubule organizing centers which appear on the surface faster to inhibit the function of T cells when T cells are activated [13]. The LAG-3 trafficking from lysosomal compartments to the cell surface is dependent on the cytoplasmic domain through protein kinase C signaling in activated T cells [14]. In areas of cholesterol-rich raft aggregation LAG-3 co-localizes with CD3 and CD4/CD8 during this primary response, as well as in the clustered raft region formed between T cells and antibody-coated beads [15]. Furthermore, some studies confirm that LAG-3 is expressed also in the cytoplasm [16,17].

Lymphocyte activation gene 3 negatively regulates T cell activation, proliferation and homeostatic expansion by regulatory T cell-dependent and independent mechanisms [18]. LAG-3 expression on a subset of regulatory T cells participates in their suppressive function [18]. Study by Annunziato et al. revealed that LAG-3 is mostly expressed in Th1, conversely Th0 and Th2 clones show weak or no LAG-3 expression [19]. What is more, LAG-3 expression on activated CD4+ subsets is correlated with higher intracellular interferon-gamma (IFN-g) production. Cytokines, such as IL-2, IL-7 and IL-12 upregulate LAG-3 expression and IL-12 is the strongest stimulus for its expression [3].

The progressive increase in LAG-3 protein expression on NK-cells correlates with time post-infection and localization to the white pulp [20]. To date, the role of regulating NK cell function is not yet clear, and therefore, calls for more research and investigation. Ali et al. suggest that LAG-3 upregulation on NK cells results in inhibitory feedback signals from surrounding MHC-II+ cells that terminate T cell suppression by NK cells [20]. In vivo studies showed that NK cells from LAG-3 deficient mice show defects in the killing of specific cancer cells [21]. However, blocking the LAG-3 pathway with LAG-3 antibodies or even soluble LAG-3 had no effect on human NK cell cytotoxicity [22,23]. Nevertheless, targeting LAG-3 may be useful in immunotherapy due to its effect on NK cell effector function [24].

CD223 was also found to be expressed in CD11c+ dendritic cells (DCs), a key cell type in the production of type I IFN [25]. Further research demonstrated that LAG-3 mRNA expression occurs in plasmacytoid dendritic cells (pDCs) and not in lymphoid or myeloid DCs [26]. Workman et al. detected LAG-3 mRNA in the red pulp of the spleen, which is a localization site for pDCs [25]. LAG-3 regulates CD11clow B220+ PDCA-1+ pDC cell homeostasis in a selective cell-intrinsic and cell-extrinsic manner. Activated pDCs may even secrete approximately five times more soluble LAG-3 than activated T cells [27]. In the presence of granulocyte-macrophage colony-stimulating factor (GM-CSF) and IL-4, LAG-3 expression results in efficient phenotypic and functional maturation and activation of human monocyte-derived DCs [28]. 

A study by Kisielow et al. shows that LAG-3 expression on activated B lymphocytes is T cells dependent and produced endogenously [29]. LAG-3 is expressed by a natural regulatory subset of plasma cells (LAG-3+CD138hi plasma cells or Bregs), which differentiate in a B cell receptor (BCR)-dependent manner [30]. LAG-3+ CD138hi plasma cells develop via an antigen-specific mechanism and present a unique epigenome poised to express IL-10 [30]. Interestingly, bruton tyrosine kinase (Btk) and BCR signaling control the development of LAG-3+ Bregs independently of Toll-like receptors (TLR) signaling and T cell help. 

Structurally CD223 is similar to the CD4 molecule and also binds to the MHC-II, but with considerably higher affinity. This connection negatively regulates T cell antigen stimulated activation, cytokine production and cytotoxicity. Connected to MHC-II, LAG-3 transmits inhibitory signals through its cytoplasmic domain and down-regulates CD4+ T lymphocytes [31]. It also interacts with the TCR complex on activated CD4 and CD8 subsets of T cells and downregulates the TCR signaling in vitro [32,33]. 

**Figure 2:**
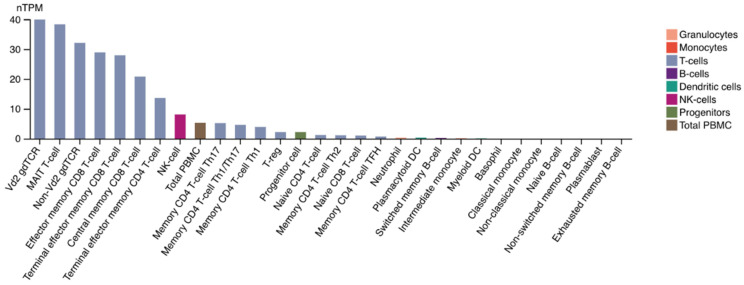
Blood cell type expression overview shows RNA-seq data generated by The Cancer Genome Atlas (TCGA). Color-coding is based on blood cell type lineages, including B-cells, T cells, NK-cells, monocytes, granulocytes and dendritic cells as well as total PBMC. Image credit: Human Protein Atlas, www.proteinatlas.org (accessed on 8 August 2022). Image available at the following URL: v21.proteinatlas.org/ENSG00000089692-LAG3/immune+cell#top.

**Figure 3:**
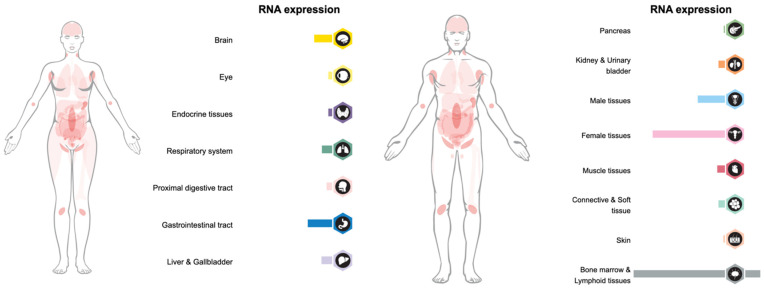
LAG-3 expression overview in tissues of the human body, visualization shows RNA-seq data generated by The Cancer Genome Atlas (TCGA) (Image credit: Human Protein Atlas www.proteinatlas.org. Image available at the following URL: v21.proteinatlas.org/ENSG00000089692-LAG3/tissue, accessed on 8 August 2022).

FGL1 is another functional LAG-3 ligand that supports its inhibitory function. The FGL1/LAG-3 interaction is highly specific. FGL1 and LAG-3 interact via the LAG-3 D1 and D2 domain and the FGL1 fibrinogen-like domain (FD) in an MHC-II-independent manner [7]. Figure 2 presents the types of immune cells expressing LAG-3, and Figure 3 provides a visual summary of the LAG-3 expression profile in body tissues.

It is important to highlight that a thorough understanding of LAG-3 expression is crucial for studies focusing on genetic modulation. In a study by Ciraolo et al., H-Y and ovalbumin antigen-specific CD8+ T cells with abolished PD-1, LAG-3, and TIM-3 expression were generated using CRISPR/Cas9 technology [34]. Genetically edited CD8+ T cells showed a strong reduction in the expression of immune checkpoint molecules after in vitro activation. This simultaneous genetic ablation of PD-1, LAG-3 and TIM-3 in CD8 T cells delayed tumor growth and improved survival. An in vivo study by Workman et al. found that mice with deficient in LAG-3, an MHC class II binding CD4 homolog, have twice as many T cells as wild-type controls [18]. Furthermore, CD4+ and CD8+ LAG-3-deficient T cells showed enhanced homeostatic expansion in lymphopenic hosts, which was abrogated by ectopic expression of wild-type LAG-3, but not by a signaling-defective mutant. A similar study showed that both the in vivo antibody blockade of LAG-3 and genetic ablation of the *LAG-3* gene resulted in an increased accumulation and effector function of antigen-specific CD8+ T cells within organs and tumors that express their cognate antigen [35]. Importantly, the combination of LAG-3 blockade with specific antitumor vaccination resulted in a significant increase in activated CD8+ T cells in the tumor and disruption of the tumor parenchyma. Interestingly, Huang et al. demonstrated that the dual blockade of antibodies against PD-1/CTLA-4 or triple blockade against PD-1/LAG-3/CTLA-4 resulted in tumor-free survival in 20% of treated mice. Conversely, dual blockade of the LAG-3 and CTLA-4 pathways using PD-1 knockout mice led to tumor-free survival in 40% of treated mice [5]. What is more, ablation of FGL1 (the major ligand of LAG-3) in mice promotes T cell immunity. Blocking the FGL1-LAG-3 interaction by monoclonal antibodies stimulates tumor immunity and is therapeutic against established mouse tumors in a receptor-ligand-dependent manner [7]. 

Excitingly, combination treatment with anti-LAG-3 and anti-PD-1 antibodies in mice resistant to treatment with single antibodies showed a strong anti-tumor effect in the absence of obvious evidence of autoimmunity, suggesting the possibility of clinical efficacy and safety through combination treatment with anti-LAG-3 and anti-PD-1 antibodies [36]. Clinical trials also confirm the findings, where in patients with advanced cancer and resistance to PD-(L)1 therapy, the use of an anti-LAG-3/PD-L1 bispecific antibody showed early signs of clinical efficacy with long-term disease control in patients with prior acquired resistance [37].

## 3. LAG-3 in Neoplasms

**Figure 4:**
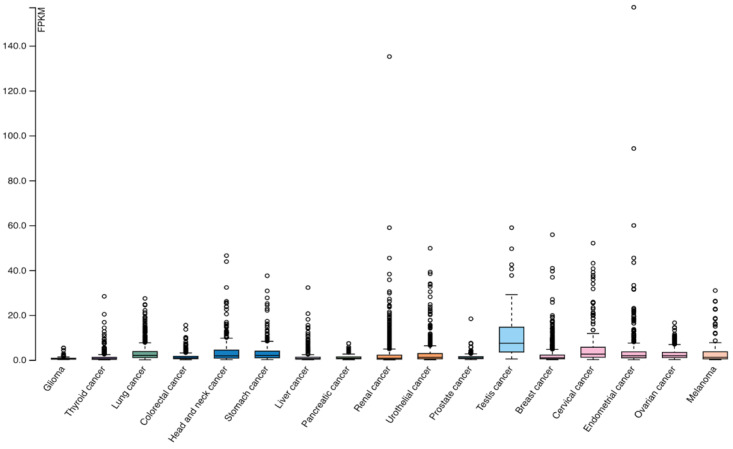
RNA-seq data across the 17 cancer types are presented as median FPKM (number of fragments per kilobase exon per million reads), generated by The Cancer Genome Atlas (TCGA). The cancer tissue RNA category is calculated based on mRNA expression levels in all 17 cancer tissues and includes enriched cancer tissues, enriched cancer groups, amplified cancer tissues, expressed in all, mixed and undetected. Normal distribution across the dataset is visualized using box plots, shown as median and 25th and 75th percentiles. Points are displayed as outliers if they are above or below 1.5 times the interquartile range. (Image credit: Human Protein Atlas, www.proteinatlas.org. Image available at the following URL: v21.proteinatlas.org/ENSG00000089692-LAG3/pathology, accessed on 8 August 2022).

Analysis of LAG-3 expression in the TCGA dataset revealed a wide expression range in various tumor types. LAG-3 modulates the tumor microenvironment by immunosuppressive effect. Differential expression in individual types of tumors indicates a different prognosis for patients [13]. Figure 4 provides a visual summary of the LAG-3 expression profile in cancer types.

### 3.1. Brain Tumors

Lymphocyte activation gene 3 is a member of the immunoglobulin superfamily of receptors expressed on microglia and neurons in the central nervous system [38,39,40,41]. Evidence suggests that in glioma LAG-3 expression is associated with CD3+, CD8+, CD20+ and PD-1+ tumor-infiltrating lymphocytes (TILs) and PD-L1+ tumor cells and hence were more frequently noted in samples with an active inflammatory microenvironment [42]. Ott et al. proved that LAG-3 levels were significantly higher in the glioma serum compared to healthy controls [43]. Overexpression of LAG-3 plays a key role in promoting tumor growth in high-grade gliomas as well as in low-grade gliomas, and its higher level predicts a prognosis of worse overall survival [44]. Thus, inhibitors of LAG-3 become attractive immune-modulating agents. A study conducted in a mouse glioblastoma model proved that LAG-3 inhibition with a blocking antibody is efficacious against glioblastoma and can be used in combination with other immune checkpoint inhibitors toward the complete eradication of model glioblastoma tumors [45]. What is more, Panda et al. found that in glioblastoma multiforme, CD8A expression was correlated with LAG-3 expression (but not with PD-L1 expression), whereas in low-grade glioma it was correlated with PD-L1 expression (but not with LAG-3 expression), suggesting that the combined blockade of LAG-3 and PD-1 may be appropriate for brain tumors with CD8+ T cell infiltration [42]. 

### 3.2. Head and Neck Tumors

In human primary head and neck squamous cell carcinoma (HNSCC), overexpression of LAG-3 on tumor infiltrating lymphocytes correlates with high pathological grade, larger tumor size and positive lymph node status. Moreover, survival analysis showed that LAG-3 may be a prognostic factor even independent of tumor size and pathological grade in patients with negative lymph node status [46]. The detection of increased LAG-3 expression in HNSCC can stratify patients into high-risk groups [47]. A study conducted by Jie et al. revealed that LAG-3 was predominantly expressed in CD4+CD25hi peripheral blood lymphocyte subgroups [48]. Similar findings were observed in a study performed by Camisaschi et. al, which proved that LAG-3 defines an active subset of CD4+CD25highFoxp3+ regulatory T cells whose frequency is increased in cancer patients and is spread at tumor sites [49]. 

### 3.3. Endocrine Tumors

Within anaplastic thyroid cancer there is a strong positive correlation between LAG-3 and PDL-1 expression, but only in male patients. Interestingly, in the group of female patients overexpression of LAG-3 did not occur [50]. Conversely, in papillary thyroid cancer most checkpoint molecules, including LAG-3, PD-1, inducible T cell COStimulator (ICOS), and indoleamine 2,3-Dioxygenase 1 (IDO1), were significantly decreased compared with healthy thyroid tissues [51]. On the other hand, in a study by Giannini et al., both anaplastic thyroid cancer and papillary thyroid cancer demonstrated an increase in immune checkpoint inhibitory mediators, including LAG-3, PDL1, PDL2, PD1, T cell immunoglobulin and mucin domain-containing-3 (TIM-3), PVR Cell Adhesion Molecule (PVR) and T cell immunoreceptor with Ig and ITIM domains (TIGIT) [52]. In the same study samples of poorly differentiated thyroid carcinomas displayed a poor or absent infiltration by immune cells. Young et al. revealed that genes associated with B cell and T cell function (LAG-3, CD27, CD28, BTLA, CTLA-4 and TIGIT) were significantly upregulated in anaplastic thyroid cancers [53]. Thus, several studies demonstrate significant differences in the immune landscape between different thyroid neoplasms. 

To date, little is known about the immune landscape of neuroendocrine tumors. The primary metastasis-like subtype showed increased expression of LAG-3, CD8B, CD38, CXCL10, CXCL9, CCL19, CD28 and CD27 compared to other subtypes [54]. In a study of 48 patients, increased LAG-3 expression was associated with markers of T cell exhaustion, while patients with upregulated CD38 and CXCL10 levels were linked to chronic infection [54].

### 3.4. Lung Tumors

LAG-3+ TILs are prevalent in two broad histological subtypes of lung cancer: small cell lung cancer (SCLC) and non-small cell lung cancer (NSCLC), which include adenocarcinoma, squamous cell carcinoma and large cell carcinoma [53]. GSE149507 cohort study revealed that LAG-3 levels were significantly overexpressed in all samples from SCLC patients compared with healthy lung tissues [55]. Similar tendencies were observed in a study conducted on 53 patients with NSCLC, where intratumoral Treg cells presented higher levels of immunosuppressive molecules, such as LAG-3, CTLA-4 and PD-1 than Tregs from tumor-adjacent tissues or peripheral blood Treg cells [56]. Moreover, it was associated with an increased number of CD4+CD25+FoxP3+ Treg cells in the peripheral blood [56]. In a study conducted by Ma et al. LAG-3-expressing CD4+CD25- T cells infiltrated the resected tumors and were more common in metastases than in primary tumors [57]. Evidence suggests that in lung tumors overexpression of LAG-3, PD-1 and TIM-3 is connected with prominent T cell activation (CD69/CD137), effector function (Granzyme-B), and proliferation (Ki-67), but also with elevated levels of proapoptotic markers (FAS/BIM) [55,58]. An increased number of Treg cells and higher expression of inhibitory molecules may have a crucial role in the anti-tumor immune response in NSCLC patients, which may contribute to tumor immune escape and tumor progression [56]. Interestingly, a study by Ding et al. demonstrated LAG-3 upregulation by measuring protein and mRNA in TILs from five of eight NSCLC patients with acquired resistance to checkpoint blockers, suggesting a possible role for LAG-3 in this setting [59]. Similarly, after treatment with PD-1 axis blockers, patients with T cell overexpression of LAG-3, showed significantly shorter progression-free survival [58]. Conversely, elevated levels of PD-1 or TIM-3 had no effect on patient survival.

In patients with lung adenocarcinoma (LUAD) higher numbers of LAG-3+ cells are correlated with features of aggressive tumor character, including predominantly solid histology, the presence of lymphovascular invasion and nodal metastases [60]. Tumors with Kirsten rat sarcoma viral oncogene homolog (KRAS) mutation demonstrate higher numbers of LAG-3+ cells than tumors with epidermal growth factor receptor (EGFR) mutation. Increased LAG-3/CD8 ratio indicates significantly worse overall survival, regardless of PD-L1 expression [57]. There is also a connection between increased expression of CTSF-associated LAG-3 and worse prognosis in patients with LUAD [61].

In lung squamous cell carcinoma (LUSC), suppressed tumoral LAG-3 expression is linked to mutations in Cut Like Homeobox 1 (CUX1), FA Complementation Group A (FANCA), or NOTCH4 genes [62]. In immune-inflamed LUSCs, *LAG-3* demonstrated a significantly higher level than other genes known to inhibit the anti-tumor immune response and was associated with poor prognosis [63].

The clinicopathological correlations and prognostic significance of LAG-3 in non-small cell lung cancer are histotype-dependent, due to differences in the immune microenvironment between adenocarcinomas and squamous cell carcinomas [64].

### 3.5. Abdominal Tumors

In gastric cancer, LAG-3 expression is positively correlated with a better prognosis [65]. In vivo experiments revealed that sLAG-3 might inhibit tumor growth, and promote the secretion of CD8+T cells, IL-12 and IFN-γ [66]. A study conducted by Ohmura et al. proved that expression of LAG-3 and CD134 on T cells promoted better prognosis in advanced gastric cancer patients treated with anti-programmed death-1 antibody [67]. Conversely, in EBV-positive and MLH1-defective gastric cancer LAG-3+ cell infiltration is strongly associated with inferior clinical outcomes [68]. There was a connection with immunoevasive contexture featured by decreased IFN-γ+ cells and perforin-1+ cells and increased levels of regulatory T cells and M2-like macrophages. 

High LAG-3 expression on tumor-infiltrating lymphocytes is positively associated with differentiation, lymph metastasis, invasion, tumor, node and metastasis (TNM) and Duke stage of colorectal cancer [69]. In stage II colorectal cancer, the expression of LAG-3 in TILs at the tumor front predicts better treatment outcomes in both the entire stage II and the subgroup of stage II microsatellite-stable tumors [70]. The immunological landscape is characteristic in each of the different cancer subtypes. Expression of LAG-3, TIM-3 and PD-1 on CD8+ CTL, Th and Treg cells is higher in colorectal cancer liver metastases than in the peritoneal metastases of colorectal cancer [71]. Colorectal cancer patients presented an enrichment of circulating Treg cells, where the LAG-3+TIM-3+ subset exhibited stronger expression of inhibitory molecules, and LAG-3+TIM-3+ Treg cells could inhibit pro-inflammatory macrophage activation [72]. However, after surgery for gastric cancer, a positive correlation was found for LAG-3 upregulation with PD-1 expression on CD4+ and CD8+ T cells, which might be related to impaired cell-mediated immunity. 

In hepatocellular carcinoma (HCC) elevated densities of LAG-3+cells and low levels of CD8+ T cells are associated with poor disease outcomes and shorter overall survival [73]. Similar tendencies were observed in immunotherapy-treated hepatocellular carcinoma, where high LAG-3 expression was associated with shorter progression-free survival [74]. Upregulated LAG-3 level in HBV-specific CD8(+) T cells is correlated with dysfunction, which leads to inhibiting HBV-specific cellular immunity in HCC [32]. In a study performed by Guo et al. high LAG-3 levels were positively associated with more cirrhosis patterns and advanced stage of cancer. Several studies confirmed that LAG-3 expression is increased in tumor-infiltrating lymphocytes relative to liver background in patients with HCC [75,76].

In pancreatic cancer high LAG-3 expression on tumor-infiltrating lymphocytes is strongly connected with PD-1 and CTLA-4 expression [77]. Due to this, the dual and triple blockade of such inhibitory receptors might improve the effectiveness of immunotherapy treatment. In pancreatic ductal adenocarcinoma (PDAC) LAG-3 expression was found to be significantly upregulated in patients with reduced disease-free survival [78]. A study by Lee et al. revealed that LAG-3 expression was highly elevated in pancreatic cancer, much more compared to other tumor types, such as hepatocellular carcinoma or gastric cancer [79]. 

### 3.6. Uro-Genital Tumors

In renal cancer, high densities of LAG-3+ cells are associated with poor prognosis [80].

It was proven that densities of DC, CD8+, PD-1+, and LAG-3+ lymphocytes in addition to PD-L1/PD-L2 expression may play a crucial role as prognostic factors [81]. Klümper et al. proved that in clear cell renal cell carcinoma (RCC) LAG-3 methylation strongly correlates with signatures of distinct immune cell infiltrates, and interferon-γ signatures and immunohistochemically quantified CD45+, CD8+, and CD4+ immune cell infiltrates [82]. Increased levels of LAG-3, methylation and tumor cell-intrinsic protein expression were correlated with overall survival. It has been noted that some patients with RCC experience a rapid increase in the number of CD4+ lymphocytes infiltrating the LAG-3+ tumor [83]. Demeure et al. found that LAG-3 expression in TILs was detected in all patients with renal cell carcinoma and varied from 11% to 48% [33]. Zhang et al. showed that the co-expression of CTLA-4, LAG-3 and TIGIT were associated with a worse prognosis in clear cell RCC, whereas in papillary RCC, the upregulation of LAG-3 with IDO1 and PD-L2 was connected with a poor outcome [84]. Moreover, LAG-3 expression is strongly correlated with the programmed cell death protein 1 gene (pdcd1) in papillary RCC, suggesting that co-targeted immunotherapy with PD-1 may induce a potent synergistic anti-tumor effect [44,85].

In testicular cancer, the overexpression of T cell markers (including LAG-3 and IFNγ) with expressed cancer/testis antigens (e.g., PRAME) in seminomas was found [86].

Higher expression of LA-G3 was also observed in SSX2 (synovial sarcoma breakpoint protein) CD8 T cells [87].

High LAG-3 expression in peripheral blood T cells and tumor-infiltrating lymphocytes correlates with histological signs of malignancy in prostate cancer [88]. However, in vivo studies showed that LAG-3 surface staining on clone 4 CD8+ cells from prostate tissue was not as apparent as LAG-3 staining from the lung, which may be caused by the presence of multiple proteases present in prostate tissue [35]. Camisaschi et al. revealed that inside the suppressor CD4+CD25highFOXP3+ T cell population, LAG-3 expression identified a subset of Tregs cells that displayed a terminal-effector phenotype and was expanded in peripheral blood and from patients with different types of cancer, among them prostate cancer [49]. It was also observed that exhausted progenitors [CD8+PD-1+TCF1+ TIM-3(−) LAG-3(−)] were the cell subtype seen at higher rates in immunogenic prostate cancer [89].

Isolated CD8+ positive tissue infiltrating lymphocytes from patients with ovarian cancer demonstrated significant upregulation in LAG-3 and high levels of PD-1 [90]. In ovarian cancer LAG-3 and PD-1 on New York Esophageal Squamous Cell Carcinoma-1 (NY-ESO-1)-specific and -nonspecific CD8+ T cells may be significantly up-regulated by tumor-derived antigen-presenting cells (APCs) or by IL-6 and IL-10 [35]. In addition, CD8+LAG-3+PD-1+ T cells were more impaired in IFN-γ/TNF-α production compared with LAG-3+PD-1- or LAG-3-PD-1- subgroups. It was found that in recurrent ovarian tumors there was a higher gene expression of LAG-3, HAVCR2 (TIM3), TIGIT and CTLA-4 than in primary tumors [91]. In vivo studies indicate that CD8+ T cells from OT-1-LAG-3−/−Pdcd1−/− mice exhibit enhanced effector function and produce more inflammatory cytokines and suggest that LAG-3 and PD-1 synergistically promote immune tolerance in ovarian tumors [92]. The results of a study conducted by Fucikova et al. reflect a strong correlation between the density of CD8+ T cells infiltrating the tumor and the number of LAG-3+, CTLA-4+ and PD-1+ cells within the tumor, suggesting that PD-1, CTLA-4 and LAG-3 behave as T cell markers in the tumor environment [93]. Indeed, we found that similar studies have shown that dual and triple antibody blockade treatment decreased the frequency of CD4+CD25+FoxP3+ cells at early time points of ovarian tumor progression [5]. Rådestad et al. proved that the proportion of CD8+ T cells without co-expression of LAG-3, TIM-3 and PD-1 is beneficial for overall survival [94]. It was also observed in the murine ovarian cancer model that LAG-3 may collaborate in recruiting SHP1 or SHP2 to the TCR complex, thereby, negatively co-regulating T-cell signaling and function [92].

The co-occurrence of LAG-3+ lymphocytes and GAL-3+ tumor cells is common in endometrial cancers, especially in tumors with methylation deficiency in mismatch repair [95]. The expression of LAG-3 is significantly higher in high-grade endometrioid carcinoma when compared to low-grade endometrioid [95]. As previously observed, LAG-3 is co-expressed with CD8A and PD-L1 in most tumor types, and overexpression is observed in endometrial cancer as well [96]. Sun et al. suggest that the aberrant expression of Lag-3 and Fgl-1 is present in the entopic and ectopic endometrium of adenomyosis and conclude that Lag-3/Fgl-1 signaling may be involved in the pathogenesis and development of adenomyosis [97]. However, it was observed that T cell exhaustion markers (LAG-3, TIM-3, TIGIT) and T cell inhibitors (PD-1, CTLA-4) were strongly correlated with CD8A expression in all endometrial cancers of all molecular subtypes [98]. 

Cervical cancer tissue samples, especially HPV-associated demonstrate high LAG-3 expression [99]. The expression of LAG-3 was detected in TILs in cervical cancer, with intensity levels ranging from 100 to 57.5%, where an association with variable PD-1 expression was also found [100]. In cervical squamous cell carcinoma and endocervical adenocarcinoma increased LAG-3 level was correlated with the co-expression of PD-1, CTLA-4 and TIM-3 [101]. In vulvar squamous neoplasia LAG-3+ tumor-infiltrating lymphocytes were identified in 91% of cases and its enhanced level was observed with GAL-3 co-expression [102]. 

### 3.7. Breast Tumors

LAG-3+ intra-epithelial tumor infiltrating lymphocytes were found in 11% of cases of breast cancer [103]. The expression of LAG-3 is positively correlated with T cells, CD8 T cells, cytotoxic lymphocytes, NK cells, B cell lineages, the monocytic lineage, and myeloid dendritic cells, but not neutrophils, endothelial cells, and fibroblasts [104]. Moreover, the higher expression of LAG-3 is associated with higher tumor grades and is enriched in the basal, HER2-positive, and luminal A (LumA) subtypes, but not in the luminal B (LumB) subtype. It was found that in triple-negative breast cancer (TNBC), LAG-3 was significantly upregulated and may be considered a potential biomarker. Another study revealed that LAG-3 correlates with glucocorticoid-induced TNF receptors across multiple tumor types [103]. In TNBC patients there was also a concurrent expression of PD-1 and LAG-3, which was observed in 15% of cases [105]. Findings suggest that the double-positive expression of LAG-3 and PD-1 predicts for a negative prognosis in breast cancer patients, affecting shortened disease-free survival, especially in patients with metastases [106]. However, Wu et al. proved that half of the PD-L1+ cases of TNBC exhibited LAG-3 co-expression [107]. In patients with a poor response to PD-1(L1) mono ICI, dual blockade of PD-1(L1) and LAG-3 may be a viable option for the treatment. The amount of LAG-3+PD-1+ T cells is different in various molecular subtypes of breast cancer, Du et al. showed that the highest expression was observed in TNBC and the lowest in ER+/PR+ breast cancer [108]. In estrogen receptor-negative breast cancers LAG-3+ iTILs demonstrated higher expression and were correlated with negative prognostic factors: young age, large tumor size, high proliferation, HER2E and basal-like breast cancer subtypes [103]. However, breast cancer patients with LAG-3+ iTILs had significantly improved breast cancer-specific survival. Similar tendencies were observed in a study performed by Stovgaard et al., wherein patients with triple-negative breast cancer LAG-3 expression was connected with relapse-free survival [109].

### 3.8. Skin Tumors

Melanoma cells expressing MHC class II attract the infiltration of tumor-specific CD4+ T cells, possibly through interactions with LAG-3, which in turn negatively affects the CD8+ T cell response [110]. In vivo studies have shown that LAG-3+ pDCs infiltrate the melanoma environment and interact with HLA-DR-expressing tumor cells. It was also found that on human plasmacytoid dendritic cells LAG-3 interacted with MHC-II to induce TLR-independent activation of pDCs with limited IFNα and enhanced IL-6 production [111]. Interestingly, LAG-3 and MHC-II interaction can downregulate T cell proliferation and protect melanoma cells from drug-induced apoptosis [112]. LAG-3-transfected cells expressing MHC class II, but not MHC class II negative, were resistant to Fas-induced apoptosis through the activation of the mitogen-activated protein kinase (MAPK) /extracellular-signal-regulated kinase (ERK) and the phosphatidylinositol 3-kinase (PI3K)/protein kinase B (AKT) survival pathways. Findings revealed that increased IL-6 stimulates the release of C-C Motif Chemokine Ligand 2 (CCL2) by monocytes in vitro, which can then recruit myeloid-derived suppressor cells (MDSCs) [113]. Andrews et al. suggest that LAG-3+ pDCs may indirectly drive MDSC-mediated immunosuppression through the engagement of MHC class II+ melanoma cells [23]. It was also shown that LAG-3+ Tregs display a terminal-effector (CD45RA+CCR7–) phenotype, produce immunosuppressive cytokines (IL-10, TGF-β1) and proliferate less than their LAG-3-negative counterparts [49]. A potential ligand that may bind to LAG-3 is liver sinusoidal endothelial cell lectin (LSECtin). An in vitro study conducted on melanoma cells demonstrated that the interaction between LAG-3 and LSECtin may inhibit IFNγ production by antigen-specific effector T cells [8]. The activation of pDCs by LAG-3 occurs at tumor sites and is partly responsible for directing the immunosuppressive environment. In uveal melanoma tumors, the levels of LAG-3 ligands-Galectin-3 and HLA class II were increased in monosomy 3 and the expression of LAG-3 correlated with the presence of an inflammatory phenotype (T cell fraction, macrophages, HLA-A and HLA-B expression) [114]. In patients with various melanomas, LAG-3 co-expression with PD-1 correlates with a state of T cell dysfunction [115,116]. The upregulation of this inhibitory receptor (IR) could mediate an escape mechanism from PD-1 therapy, in which resistance might possibly be overcome with the addition of LAG-3 blockade. Similarly, antigen-specific T cells (Melan-A/MART-1) extracted from metastases of melanoma patients exhibit increased levels of LAG-3 and other IRs (CTLA4, TIM3) compared with the expression on peripheral blood lymphocytes [117]. The LAG-3 expression may play a role as a predictive marker; in LAG-3 positive patients the response was significantly higher than in patients with a LAG-3 presence of less than 1% of positive tumor cells, the response rate equaled 20% vs. 7% [118]. What is more, a study by Shen et al. classified the phenotypes and determined their relationship to survival and response to treatment [119]. Patients with melanoma with a LAG+ immunotype had poorer outcomes after immune checkpoint blockade with a median survival of 22.2 months compared to 75.8 months for those with the LAG− immunotype. A study by Machiraju et al. revealed that in patients treated with PD-1 monotherapy, increased levels of sLAG3 in pre-treatment samples were observed in resistant cases [120]. Patients with increased serum sLAG3 levels had significantly shorter progression-free survival after therapy.

### 3.9. Lymphoid Tumors

In Hodgkin’s lymphoma (HL), malignant Hodgkin Reed–Sternberg (HRS) cells make up only 0.5% of 10% of diseased tissue, and the surrounding cellular infiltrate is enriched with T cells that may modulate anti-tumor immunity [121]. High LAG-3 expression on tumor infiltrating lymphocytes (TILs) and peripheral blood lymphocytes (PBLs) is associated with the suppression of EBV-specific T cell function [32]. CD4+ LAG-3 circulating regulatory T cells were significantly elevated in HL patients with active disease compared with remission. LAG-3, are nearly always co-expressed with TIM-3 in the microenvironment of classical HL [122]. However, a study conducted on 57 biopsy samples of patients with classical HL revealed that LAG-3 expression in ≥5% of HRS cells was detected in only 5.2% of cases [122]. Conversely to TIM-3–positive HRS cells, where >36.2% of tissue samples had >5% expression. 

In non-Hodgkin lymphoma, the development of T cell exhaustion is defined by LAG-3, PD-1 and TIM-3 expression [123].

In follicular lymphoma overexpression of LAG-3 is associated with poor clinical outcomes. Yang et al. found that LAG-3 was expressed on a subset of intertumoral T cells from follicular lymphoma and LAG-3+ T cells almost exclusively came from the PD-1+ population [124]. LAG-3 expression was substantially upregulated on CD4+ or CD8+ T cells by IL-12 (a cytokine increased in the serum of lymphoma patients that induces T cell exhaustion). Interestingly, blockade of both PD-1 and LAG-3 signaling improved the function of intra-neoplastic CD8+ T cells, resulting in the increased production of IFN-γ and IL-2.

## 4. Anti-LAG-3 Antibody-Based Therapies

LAG-3 targeting therapies can be divided into three subtypes: anti-LAG-3 monoclonal antibodies, bispecific LAG-3 and LAG-3 (Ig) immunoglobulin fusion proteins. Anti-LAG-3 monoclonal antibody (mAb) inhibits both IL-12 and IFN-γ production in IL-2-stimulated cocultures of T cells and autologous monocytes. Blocking LAG-3/MHC contact using an anti-LAG-3 mAb not only suppresses the positive signal given to monocytes via MHC class II but also inhibits T cell response to IL-12 [125]. However, it is increasingly recognized that LAG-3 blockade alone may not be an ideal treatment strategy. This can be explained by the ability of cancer cells to evade anti-tumor immune responses with countless molecules.

Due to quite flexible pathways of functioning, bispecific antibodies (BsAbs) have become a new subject of future research and medical development. BsAbs are antibodies with two binding sites targeting two different antigens or two different epitopes on the same antigen [126]. With the exception of antibody fragments, full-length bispecific antibodies with Fc-mediated immune activity show greater potential for anti-tumor immunotherapy. The combination of anti-LAG-3 and anti-PD-1 therapies, evaluated in clinical trials can significantly enhance the anti-tumor effect [37]. LAG-3 is co-expressed with PD-1 on tumor-infiltrating CD8+ T cells, and the co-blockade of PD-1 and LAG-3 by antibodies elevated CD8+ T cells proliferation and cytokine production [127]. The study of Blackburn et al. showed encouraging results in a murine model of chronic viral infection. It was proved that the dual blockade of LAG-3 and PD-1 in vivo resulted in significant increases in antigen-specific CD8+ T cell numbers and function, as well as marked reductions in viral titer [128]. The combination of anti-PD-1 and anti-LAG-3 therapy may be beneficial in Hodgkin lymphoma (HL). Nagasaki et al. showed that combination treatment with anti-PD-1 mAb and anti-LAG-3 mAb exhibited far stronger antitumor efficacy on MHC-II–expressing tumors than either mAb alone. LAG-3 inhibits the anti-tumor effect of anti-PD-1 and anti-LAG3 therapy in HL by inhibiting the CD4+ T cell responses; chemotherapy with ABVD (doxorubicin, bleomycin, vinblastine, and dacarbazine) shows little therapeutic effect with a higher infiltration of LAG-3+TILs [129]. 

IMP321 is the only soluble recombinant LAG-3 clinically studied. Molecule IMP321 is a fusion protein consisting of LAG-3 extracellular domains fused to a human immunoglobulin Fc region, obtained by replacing the Fab immunoglobulin domains of an IgG1 with the four immunoglobulin-like domains from the extracellular region of LAG-3. IMP321 is a nontypical immune checkpoint inhibitor as it activates antigen-presenting cells (APCs) through interaction with MHC-II, which is present on their membrane. LAG-3-Ig interaction with MHC class II on human immature DCs induced the up-regulation of CD80/CD86, secretion of IL-12 and TNFα, and promoted morphological changes, such as the formation of dendritic projections. Hence, IMP321, by enhancing APC activation, acts differently from antagonist LAG-3 antibodies which block the LAG-3 / MHC-II interaction and thus LAG-3 mediated downregulation of T cells [2,130]. 

**Figure 5:**
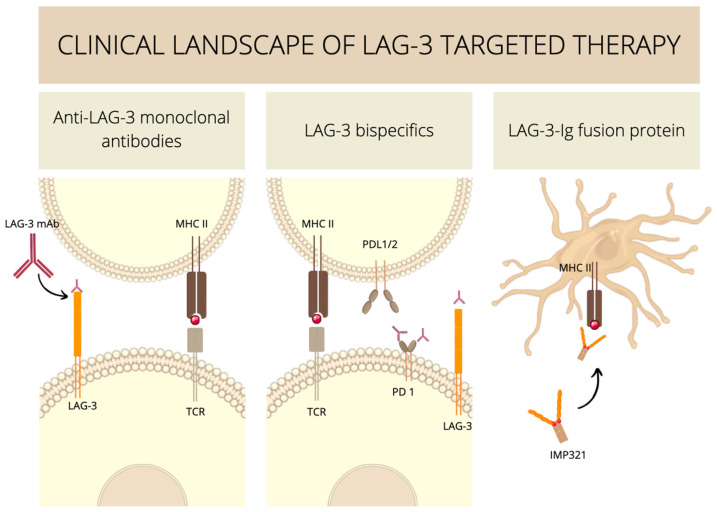
Schematic representation of LAG-3-targeted therapies: anti-LAG-3 monoclonal antibodies, and bispecific LAG-3, LAG-3 (Ig) immunoglobulin fusion proteins.

IMP321 clinical efficacy was minimal as a monotherapy, but there have been more promising effects with IMP321 when combined with cytotoxic chemotherapies and vaccine-based strategies. Lawrence P Andrews et al. describe a trial where melanoma patients were treated with melanoma antigen recognized by T cell 1 (MART-1) peptide vaccination, with or without IMP321. The point was to investigate the potential synergy of adoptive T cell transfer and immunomodulation. As a result, the analysis of MART-1 specific CD8 + T cells among IMP321-treated patients showed reduced expression of depletion markers including PD-1, LAG-3, TIM-3, CD244 and CD160. Moreover, in addition to the improved responses and functionality of antigen-specific cytotoxic T lymphocytes (CTLs), immunization with IMP321 selectively inhibited Treg expansion, suggesting that the relative increase in the CD8 + to Treg effector ratio may partially explain the beneficial immune responses observed with IMP32 [23]. Figure 5 summarizes LAG-3-targeted therapies.

## 5. Anti-LAG-3 Cell-Based Therapies

In recent years, genetically modified immune cells, in particular chimeric antigen receptor T (CAR-T), have generated great interest in clinical research as a promising treatment for cancer.

Zhang et al. generated LAG-3 knockout T and CAR-T cells by not changing the viability and immune phenotype during in vitro culture. To achieve the effect they used Clustered Regularly Interspaced Short Palindromic Repeats (CRISPR) and the CRISPR-associated protein (Cas) (CRISPR-Cas9) system. The delivery of the CRISPR-Cas9 system via electroporation provides an efficient platform to knockout LAG-3 in T and CAR-T cells which are rich in the central memory subtype. LAG-3 knockout CAR-T cells maintained their antigen-specific cytokine release and anti-tumor potency in vitro and in vivo [131].

Blockade of PD-1 in combination with other immune checkpoint receptors, including LAG-3, has strong synergistic effects and boosts the effector functions of CAR-T cells. The high efficacy of this combinatorial approach suggests that LAG-3 pathways have non-redundant effects that synergize with PD-1 signaling to dampen antitumor responses in dysfunctional CAR T cells [132].

Elisa Ciraolo et al. aimed to demonstrate that genetic editing of PD-1, LAG-3, and TIM-3 is not harmful to the functionality of CD8+ T cells. The data demonstrate that the simultaneous genetic ablation of PD-1, LAG-3, and TIM-3 expression induced a more sustained anti-tumor activity compared to non-edited T cells resulting in reduced tumor growth as well as increased survival. MGD013 demonstrates in vitro ligand blocking properties and improved T cell responses beyond that observed with anti-PD-1 and anti-LAG-3 benchmark antibodies alone or in combination [133].

## 6. Experimental Medicine Involving LAG-3 Clinical Trials

Several clinical trials revealed the expression of the CD15 molecule in pathology conditions. Therapies include monoclonal antibodies, soluble LAG-3–immunoglobulin (Ig) fusion proteins and anti-LAG-3 bispecific drugs. According to ClinicalTrials.gov, there are currently 115 studies conducted across the world investigating the safety and efficiency of these drugs. The majority of anti-LAG-3 monoclonal antibodies are fully humanized IgG4-blocking monoclonal antibodies. IMP321 is the only soluble recombinant LAG-3 under clinical investigation. In addition, studies on bi-specific drugs directed against LAG-3 are ongoing and results are very promising, especially on PD-1/LAG-3 blockade.

### 6.1. Anti-LAG-3 Monoclonal Antibodies

Relatlimab (BMS-986016) is a novel LAG-3 blocking antibody currently being evaluated in 48 clinical trials. Treatment in monotherapy reduces the number of leukemic cells and restores NK and T cell-mediated responses and promotes T cell tumor necrosis factor (TNF)-α, IFN-γ and IL-2 cytokines [134]. Combination therapy with the lenalidomide (immunomodulatory drug) increased IL-2 production by T cells and antibody-dependent cytotoxicity (ADCC) mediated by NK cells. Relatlimab and nivolumab in combination provided a greater benefit with regard to progression-free survival than the inhibition of PD-1 alone in patients with previously untreated metastatic or unresectable melanoma [135]. Moreover, the same drug combination in patients with gastric or gastroesophageal junction adenocarcinoma is currently being evaluated in the recruitment phase (NCT03662659).

Fianlimab (REGN3767) is a high-affinity, fully human, hinge-stabilized IgG4 monoclonal antibody. By blocking the binding of LAG-3 to MHC class II, fianlimab activates T cells and enhances tumor cell lysis mediated by cytotoxic T cells. Combination therapy with the cemiplimab (PD-1 blocking antibody) and fianlimab showed increased efficacy (in vitro and in a mouse tumor model) and enhanced the secretion of proinflammatory cytokines by tumor-specific T cells [136]. The safety profile of R3767 with cemiplimab was generally tolerable and early efficacy signals were detected despite the difficult-to-treat population [137]. In patients with advanced melanoma, the fianlimab and cemiplimab combination has shown clinical activity that is similar to anti-PD-1 and CTLA-4 combination therapy, but with lower demonstrated rates of treatment-emergent adverse events [138]. There are five clinical trials investigating REGN3767 in monotherapy and in combination with anti-PD-1 inhibitors.

89Zr-DFO-REGN3767 (fianlimab tracer) is comprised of the anti-LAG-3 antibody, REGN3767 labeled with the positron-emitter zirconium-89 (89Zr) through the chelator-linker. Antibodies labeled with radioactive isotopes are essential in the development of diagnostic and radiotherapeutic agents for PET or radioimmunotherapy [139]. 89Zr-DFO-REGN3767 is currently under evaluation in two clinical trials to monitor response to anti-LAG-3 drug therapy. The main objective of the clinical trials is to better understand how the body absorbs, distributes and disposes of 89Zr-DFO-REGN3767, to find the best dose and the best time to perform a PET scan after injection.

Sym022 is an Fc-inert human monoclonal antibody that binds to human LAG-3 with high affinity and blocks the interaction between LAG-3 and MHC-II molecules. Sym022 enhances cytokine production by T cells in vitro and inhibits tumor growth in vivo [140]. Moreover, treatment with Sym022 lowers the total surface level of LAG-3 by internalization and/or shedding. Three clinical trials are investigating Sym022 alone and in combination with Sym021 (anti-PD-1) and Sym023 (anti-TIM-3) (NCT03489369, NCT04641871, NCT03311412). Preliminary data showed that Sym021 monotherapy was well tolerated and exhibited both immune modulation and anti-tumor activity, while in combination with Sym021 and Sym023 there was a synergistic anti-tumor effect [141]. Data from clinical trials of Sym022 in patients with advanced malignant solid tumors or lymphomas showed no serious adverse drug reactions after the first and second doses. The third dose caused chest pain in one in three patients, and the fourth dose caused gastrointestinal hemorrhage, increased lipase levels and tumor pain in one in six patients.

GSK2831781 (IMP731) is an afucosylated humanized IgG1 monoclonal antibody enhanced with a high affinity for Fc receptors and LAG-3 and antibody-dependent cellular cytotoxicity capabilities [142]. In a double-blind, placebo-controlled phase I/Ib clinical trial, treatment with GSK2831781 at doses ≥ 0.15 mg/kg resulted in the depletion of LAG-3+ cells in peripheral blood and was dose-dependent [143]. After treatment with a 5 mg/kg dose of GSK2831781, there was a decrease in the expression of pro-inflammatory genes (IL-17A, IL-17F, IFNγ and S100A12) and increased expression of the epithelial barrier integrity gene, CDHR1. Moreover, a study by Slevin et al. revealed that GSK2831781 treatment led to a depletion of LAG-3+CD4+ and CD8+ T cells [144].

INCAGN02385 is an Fc-engineered IgG1κ monoclonal antibody with the ability to potently block LAG-3 binding with its MHC class II ligand. INCAGN02385 increases T cell reactivity to TCR stimulation, both in monotherapy and in combination with anti-PD-1/PD-L1 drugs. The INCAGN02385 treatment in cynomolgus monkeys was well tolerated and presented a safe pharmacokinetic profile [145]. There are currently three clinical trials for the treatment of advanced malignancies (NCT03538028, NCT04370704, NCT05287113).

TSR-033 is a high affinity and selective humanized monoclonal IgG4 antibody that binds human LAG-3 and serves as a functional antagonist. In vitro TSR-033 enhances T cell activation in mixed lymphocyte reactions and staphylococcal enterotoxin B-driven stimulation assays [146]. Studies using mouse replacement antibodies proved that the combined blockade of PD-1 and LAG-3 significantly enhanced IFNγ production in an in vitro model of CD4 T cell depletion in mice [146]. Sullivan et al. found that the inhibition of LAG-3 with TSR-033 resulted in a significant increase in the calcium fluctuations of CD8+ T cells in contact with dendritic cells and in combination with TSR-042 (anti-PD-1), synergistically enhanced tumor cell killing at the single-cell level [147]. Moreover, in ovarian cancer the triple combination of TSR-033, TSR-042 (anti-PD-1), and TSR-022 (anti-TIM-3) stimulated cytokine release, indicating a more efficient activation of T lymphocytes that infiltrate tumors [146]. TSR-033 is currently being evaluated in two clinical trials in the recruiting phase for the treatment of advanced solid tumors.

LAG525 (Ieramilimab) is a humanized IgG4 monoclonal antibody, which blocks LAG-3 binding to MHC-II. Five clinical trials are evaluating LAG525 at various clinical stages. LAG525 in combination with spartalizumab was well tolerated with preliminary anti-tumor activity in a variety of solid tumors, including mesothelioma and triple-negative breast cancer, neuroendocrine tumors (NET), small cell lung cancer (SCLC) and diffuse large B-cell lymphoma (DLBCL) [148,149]. Patients treated with LAG525 and spartalizumab initially demonstrated higher levels of expression of immune genes, including CD8 and LAG-3, in the tumor tissue [150]. Spartalizumab administered with LAG525 achieved an astounding 86% of clinical benefit rate at 24 weeks in a gastroenteropancreatic neuroendocrine tumors cohort [149]. In this combination, no new safety signals were observed, and the toxicity profile of ieramilimab in combination with spartalizumab was comparable to monotherapy of spartalizumab [151].

MK-4280 (favezelimab) is a humanized anti-LAG-3 monoclonal antibody that blocks the interaction between LAG-3 and its ligand MHC class II. MK-4280 treatment increases the production of cytokines, such as IFN-γ, IL-2, IL-8 and TNF-α and chemokines (CCL4, CXCL10 and CCL22) in T cells, moreover, CD69, CD44, and CD25 were up-regulated [152]. Preclinical oncology studies support the concept of co-targeting LAG-3 to increase the therapeutic efficacy of PD-1 blockade. Currently, MK-4280 in combination with pembrolizumab demonstrated anti-tumor activity across several syngeneic mouse tumor models and is under evaluation as a first-line therapy for patients with advanced renal cell carcinoma [153,154]. Preliminary results demonstrated good safety and efficacy profiles both in monotherapy and in combination therapy [155].

The examples of clinical trials involving Anti-LAG-3 Monoclonal Antibodies are shown in Table 1.

### 6.2. Anti-LAG-3 Bispecifics

Tebotelimab (previously known as MGD013) is a bispecific antibody that targets the programmed cell death-1 (PD-1) receptor and lymphocyte activation gene (LAG-3) and chronically activated T cells. Following MGD013 treatment, serum IFN-γ levels increased significantly and an expansion of circulating CD3+CD8+ and CD3+CD4-CD8- T cell subpopulations and associated cytolytic markers (i.e., perforin, granzyme B) was observed [133]. There are seven clinical trials evaluating MGD013 monotherapy and combinations. MGD013 monotherapy showed antitumor activity in multiple tumor types, such as melanomas and advanced hepatocellular carcinoma (HCC) (Table 2). Combined therapy involving MGD013 with margetuximab revealed positive results in patients with HER2+ breast cancer [156]. A phase I clinical trial tested MGD013 in patients with relapsed or refractory DLBCL, demonstrating good pharmacodynamics, safety profile and anti-tumor activity in combination with and without prior CAR-T cell treatment [133].

RO7247669 is an anti-PD-1/anti-LAG-3 bispecific antibody, which targets and binds to both PD-1 and LAG-3 expressed on T cells and inhibits the PD-1- and LAG-3-mediated downregulation of T cell activation and proliferation. These lead to a cytotoxic T lymphocyte (CTL)-induced immune response against tumor cells. RO7247669 is currently being evaluated in four clinical trials in the recruitment phase.

FS118 is a bispecific antibody against LAG-3 and PD-L1 with the potential to reinvigorate exhausted immune cells and overcome resistance mechanisms to PD-L1 blockade. It was proven that FS118 simultaneously bound to LAG-3 and PD-L1, blocked PD-1/PD-L1, CD80/PD-L1, and LAG-3/MHC-II interactions and as a result, reversed T cell inhibition. FS118 boosts cytokine production by CD4+ and CD8+ T cells, which play a key role in the anti-tumor response [157]. Its mouse surrogate version has been shown to promote the removal of LAG-3 and PD-L1 through the activities of disintegrin and metalloproteinase 10, and a disintegrin and metalloproteinase 17. Preliminary data suggest good tolerability and early signs of clinical efficacy with long-term disease control [158]. FS118 is currently being evaluated in one clinical trial in the recruitment phase for the treatment of advanced malignancies.

EMB-02 is a bispecific antibody, designed to simultaneously target human PD-1 and LAG-3 and interfere with the immune suppression mediated by both pathways, thereby restoring effector T cell function and enhancing anti-tumor activity. There is only one clinical trial at the recruitment stage in selected advanced solid tumors.

IBI323 is a dual blockade bispecific antibody targeting PD-L1 and LAG-3 with similar potency as its parental antibodies. IBI323 blocks PD-1/PD-L1, CD80/PD-L1, and LAG-3/MHC-II interactions. In PD-L1/LAG-3 double knock-in mice carried human PD-L1 MC38 tumors, IBI323 exhibited more potent anti-tumor effects in comparison with each of the parental antibodies [159]. Treatment with IBI323 correlated with increased tumor-specific CD8+ and CD4+ T cells [159]. One clinical trial is currently recruiting patients to evaluate IBI323 in advanced solid malignancies.

XmAb841 is an anti-CTLA4-LAG-3 bispecific antibody with a modified Fc domain that increases the stability and long-circulating half-life of the antibody [37]. XmAb22841 targets and binds to CTLA-4 and LAG-3 expressed on T cells in the tumor microenvironment. Both CTLA-4 and LAG-3 are inhibitory receptors that belong to the immunoglobulin superfamily (IgSF) overexpressed on regulatory T cells (Tregs) in the tumor microenvironment, where they inhibit T cell activation and proliferation. This bispecific compound can be combined with pembrolizumab (anti-PD-1 antibody) to promote triple checkpoint blockade and increase allogeneic anti-tumor activity (NCT03849469). 

The examples of clinical trials involving Anti-LAG-3 bispecifics are shown in Table 2.

### 6.3. Soluble LAG-3–Ig Fusion Proteins

Eftilagimod alpha known as IMP321 or efti is a soluble version of the immune checkpoint molecule LAG-3. IMP321 is an unusual immune checkpoint inhibitor as it targets antigen-presenting cells, and transduces an MHC-II-mediated feedback signal. IMP321 increased T cell proliferation and induced a full Tc1-activated phenotype characterized by the production of IFN-γ, TNF-α, IL-1β, IL-6, CCL4, CCL5, and CCL2. Moreover, IMP321 treatment promotes myeloid cells to produce CCL4 and TNF-α, and CD8 and NK cells to produce IFN-γ and TNF-α [160]. Soluble LAG-3 fusion protein increases the capacity of phagocytic cells (MHC class II1 macrophages or immature dendritic cells (DCs)) to induce T cell responses. IMP321 induces tumor regression and antitumor immune responses involving the recruitment of a CD8(+) T cell response. There are currently 13 clinical trials involving LAG-3-IG fusion protein. Moreover, IMP321 demonstrates safety, tolerability and good efficacy in combination with other therapies [161]. The lack of toxicity and the demonstration of activity strongly support the future development of this drug for clinical use in combination with first-line regimens. The examples of clinical trials involving soluable LAG-3-Ig fusion proteins are shown in Table 3.

## 7. Conclusions and Perspectives

LAG-3, an inhibitory immune checkpoint, is considered a highly promising target in novel cancer treatment strategies and has been attracting great research interest in recent years as a tool potentially enabling to overcome the certain limitations of conventional therapies. Although significant progress in the field of cancer immunotherapy has been made, the understanding of LAG-3 functional properties is still limited and needs further investigation to be completely evaluated. In this manuscript, we have reviewed the current state of knowledge on LAG-3 structure, function, and expression in different cancer types along with its correlation to overall prognosis and its involvement in cell-based therapies and experimental medicine. We have shown that LAG-3 targeting compounds are extensively studied in clinical trials both in monotherapy and combination, which would provide data referring to the efficacy and safety of the proposed drug candidates. In light of the results of reviewed studies, both in vivo and in vitro on cell cultures, it is evident that LAG-3 could be a great target for therapeutic intervention, but there are still many uncertainties.

Undoubtedly, obtaining more detailed knowledge of LAG-3 functional properties would allow for the design and development of new generation therapeutic strategies targeted toward various tumor types. Therefore, further research is necessary to address issues concerning LAG-3 biology and function, such as the existence of other LAG-3 ligands along with their expression, exact signaling pathways through which LAG-3 influence on TCR function is exerted, or mechanisms underlying LAG-3/PD-1 synergy. The question which also needs to be answered is whether LAG-3 could be considered a cancer clinical biomarker, as has been suggested in several reports. Moreover, as a part of the long-term future perspective, the identification of different types of cancer which would most likely respond to specific combined immunotherapy dependent on several factors, such as their microenvironment or antigens presented, would create a possibility to apply a personalized therapeutic approach towards each cancer patient.

## Figures and Tables

**Table 1:** Examples of Clinical Trials involving Anti-LAG-3 monoclonal antibodies.

Identifier	Patients Number	Recruitment Status	Condition or Disease	Target	Therapy Protocol	Short Description
NCT01968109	1499	Active, not recruiting	Neoplasms by Site	LAG-3 PD-1	Biological: Relatlimab Biological: Nivolumab Biological: BMS-986213	Anti-LAG-3 Monoclonal Antibody (BMS-986016) Administered Alone and in Combination with Anti-PD-1 Monoclonal Antibody (Nivolumab, BMS-936558) in Advanced Solid Tumors
NCT03662659	274	Active, not recruiting	Gastric Cancer Cancer of the Stomach Esophagogastric Junction	LAG-3 PD-1	Biological: BMS-986213 Biological: Nivolumab Drug: XELOX Drug: FOLFOX Drug: SOX	Relatlimab and Nivolumab in Combination with Chemotherapy Versus Nivolumab in Combination with Chemotherapy as First-Line Treatment in Patients with Gastric or Gastroesophageal Junction Adenocarcinoma
NCT02061761	107	Active, not recruiting	Hematologic Neoplasms	LAG-3 PD-1	Biological: BMS-986016 Biological: BMS-936558	Anti-LAG-3 Monoclonal Antibody (Relatlimab, BMS-986016) Administered Alone and in Combination with Anti-PD-1 Monoclonal Antibody (Nivolumab, BMS-936558) in Relapsed or Refractory B-Cell Malignancies
NCT03493932	20	Active, not recruiting	Glioblastoma	LAG-3	Drug: Nivolumab Drug: BMS-986016	Nivolumab, together with an anti-LAG-3 antibody BMS-986016 in Patients with Glioblastoma
NCT04150965	104	Active, Recruiting	Multiple Myeloma Relapsed Refractory Multiple Myeloma	LAG-3 TIGIT	Drug: Elotuzumab, pomalidomide, dexamethasone Drug: Anti-LAG-3 Drug: Anti-LAG-3 + Pomalidimide + Dexamethasone Drug: Anti-TIGIT Drug: Anti-TIGIT + Pomalidimide + Dexamethasone	Combination Immuno-Oncology Drugs Elotuzumab, Anti-LAG-3 (BMS-986016) and Anti-TIGIT (BMS-986207) in Patients with Multiple Myeloma
NCT03044613	32	Active, not recruiting	Gastric Cancer Esophageal Cancer Gastroesophageal Cancer	LAG-3 PD-1	Drug: Nivolumab Drug: Relatlimab Drug: Carboplatin Drug: Paclitaxel Radiation: Radiation	Nivolumab or Nivolumab/Relatlimab Prior to Concurrent Chemoradiation in Patients with Operable Stage II/III Esophageal/ Gastroesophageal Junction Cancer
NCT04080804	60	Active, Recruting	Head and Neck Squamous Cell	LAG-3 PD-1 CTLA-4	Drug: Nivolumab Drug: Relatlimab	Anti-PD1 (Nivolumab) Administered Alone or in Combination with Anti-LAG3 (Relatlimab) or Anti-CTLA4 (Ipilimumab) in Resectable Head and Neck Cancer
NCT03459222	255	Active, Recruiting	Advanced Cancer	LAG-3 PD-1 CTLA-4	Biological: Relatlimab Biological: Nivolumab Drug: BMS-986205 Biological: Ipilimumab	Relatlimab (Anti-LAG-3 Monoclonal Antibody) Administered in Combination with Both Nivolumab (Anti-PD-1 Monoclonal Antibody) and BMS-986205 (IDO1 Inhibitor) or in Combination with Both Nivolumab and Ipilimumab (Anti-CTLA-4 Monoclonal Antibody) in Advanced Malignant Tumours
NCT03005782	669	Active, Recruting	Malignancies	LAG-3 PD-1	Drug: REGN3767 Drug: cemiplimab	REGN3767 (Anti-LAG-3 mAb) Administered Alone or in Combination with REGN2810 (Anti-PD-1 mAb) in Patients with Advanced Malignancies
NCT04566978	20	Active Recruiting	Large B-cell Lymphoma DLBCL	LAG-3	Drug: 89Zr-DFO-REGN3767 Diagnostic Test: PET/CT	89Zr-DFO-REGN3767 Anti LAG-3 Antibody Positron Emission Tomography in Patients with Relapsed/Refractory DLBCL
NCT03489369	15	Completed, Phase 1	Metastatic Cancer Solid Tumor Lymphoma	LAG-3	Experimental: Sym022	Antineoplastic Activity of Sym022 (Anti-LAG-3) in Patients with Advanced Solid Tumor Malignancies or Lymphomas
NCT03311412	91	Completed, Phase 1	Metastatic Cancer Solid Tumor Lymphoma	LAG-3, PD-1, TIM-3	Drug: Sym021 Drug: Sym022 Drug: Sym023	Activity of Sym021 (Anti-PD-1) as Monotherapy, in Combination with Either Sym022 (Anti-LAG-3) or Sym023 (Anti-TIM-3), and in Combination with Both Sym022 and Sym023 in Patients with Advanced Solid Tumor Malignancies or Lymphomas
NCT04641871	100	Active Recruiting	Metastatic Cancer Solid Tumour	LAG-3 PD-1 TIM-3	Drug: Sym021 Drug: Sym022 Drug: Sym023 Drug: Irinotecan Hydrochloride	Sym021 (Anti-PD 1) in Combination with Either Sym022 (Anti-LAG-3) or Sym023 (Anti-TIM-3) or Sym023 and Irinotecan in Patients with Recurrent Advanced Biliary Tract Carcinomas
NCT03250832	111	Active, not recruiting	Neoplasms	LAG-3 PD-1	Drug: TSR-033 Drug: Dostarlimab Drug: mFOLFOX6 Drug: FOLFIRI Drug: Bevacizumab	TSR-033, an Anti-LAG-3 Monoclonal Antibody, Alone and in Combination with an Anti-PD-1 in Patients with Advanced Solid Tumours
NCT03499899	88	Completed	Triple-negative Breast Cancer	LAG-3 PD-1	Drug: LAG525 Drug: spartalizumab Drug: carboplatin	LAG525 in Combination with Spartalizumab, or with Spartalizumab and Carboplatin, or with Carboplatin, in Patients with Advanced Triple-negative Breast Cancer
NCT02460224	490	Completed	Advanced Solid Tumours	LAG-3 PD-1	Drug: LAG525 Drug: PDR001	LAG525 Single Agent and in Combination with PDR001 Administered to Patients with Advanced Malignancies
NCT03484923	196	Active, not recruiting	Melanoma	LAG-3 PD-1 MET IL-1β CDK4/6	Drug: Spartalizumab Drug: LAG525 Drug: Capmatinib Drug: Canakinumab Drug: Ribociclib	Spartalizumab (PDR001) Combinations in Previously Treated Unresectable or Metastatic Melanoma
NCT05064059	432	Active, recruiting	Colorectal Cancer	LAG-3 PD-1	Biological: favezelimab/pembrolizumab Drug: regorafenib Drug: TAS-102	Favezelimab/Pembrolizumab (MK-4280A) in participants with metastatic colorectal cancer
NCT03598608	154	Active, recruiting	Hodgkin Disease Lymphoma, Non-Hodgkin Lymphoma, B-Cell	LAG-3 PD-1	Biological: pembrolizumab Biological: Favezelimab	Combination of MK-4280 and Pembrolizumab (MK-3475) in Participants with Hematologic Malignancies

**Table 2:** Examples of Clinical Trials involving Anti-LAG-3 bispecifics.

Identifier	Patients Number	Recruitment Status	Condition or Disease	Target Antigen	Therapy Protocol	Short Description
NCT03219268	353	Active, not recruiting	Advanced Solid Tumors Hematologic Neoplasms Ovarian Cancer HER2-positive Advanced Solid Tumors Non-Small Cell Lung Cancer Small-cell Lung Cancer Squamous Cell Carcinoma of Head and Neck Cholangiocarcinoma Cervical Cancer TNBC-Triple-Negative Breast Cancer	LAG-3 PD-1	Biological: tebotelimab Biological: margetuximab	MGD013, A Bispecific DART^®^ Protein Binding PD-1 and LAG-3 in Patients with Unresectable or Metastatic Neoplasms
NCT04140500	320	Active, Recruiting	Solid Tumors Metastatic Melanoma Non-small Cell Lung Cancer Esophageal Squamous Cell Carcinoma	LAG-3 PD-1	Drug: RO7247669	RO7247669, a PD1-LAG3 Bispecific Antibody, in Patients with Advanced and/or Metastatic Solid Tumours
NCT03440437	80	Active, Recruiting	Advanced Cancer Metastatic Cancer Squamous Cell Carcinoma of Head and Neck	LAG-3 PD-1	Drug: FS118	FS118, a LAG-3/PD-L1 Bispecific Antibody, in Patients with Advanced Malignancies
NCT04618393	43	Active, Recruting	Advanced Solid Tumor	LAG-3 PD-1	Biological: EMB-02	EMB-02, a Bi-specific Antibody Against PD-1 and LAG-3, in Patients with Advanced Solid Tumors
NCT04916119	322	Active Recruiting	Advanced Malignancies	LAG-3 PD-1	Drug: IBI323	IBI323(anti-LAG-3/PD-L1) or in combination with chemotherapy in participants with advanced malignancies
NCT03849469	242	Active Recruiting	Solid tumors	LAG-3 CTLA-4	Biological: XmAb^®^22841 Biological: Pembrolizumab (Keytruda^®^)	XmAb22841 monotherapy and in combination with pembrolizumab in Patients with Solid tumors

**Table 3:** Examples of Clinical Trials involving Soluble LAG-3-Ig fusion proteins.

Identifier	Patients Number	Recruitment Status	Condition or Disease	Target Antigen	Therapy Protocol	Short Description
NCT00349934	33	Completed, Phase 1	Metastatic Breast Cancer	LAG-3	Biological: IMP321	IMP321 in Metastatic Breast Carcinoma Patients Receiving First-line Paclitaxel
NCT02614833	242	Completed	Adenocarcinoma Breast Stage IV	LAG-3	Biological: IMP321 (eftilagimod alpha) Drug: Placebo Drug: Paclitaxel	Study in Hormone Receptor-positive Metastatic Breast Carcinoma Patients Receiving IMP321 (LAG-3Ig Fusion Protein) or Placebo as Adjunctive to a Standard Chemotherapy Treatment Regimen of Paclitaxel
NCT00351949	24	Completed	Stage IV Renal Cell Carcinoma	LAG-3	Biological: IMP321	IMP321 in Advanced or Metastatic Renal Cell Carcinoma Patients
NCT03252938	45	Active, Recruiting	Solid Tumors Peritoneal Carcinomatosis	LAG-3	Drug: IMP321 Drug: Avelumab	MP321 (LAG-3Ig Fusion Protein) in Patients with Advanced Stage Solid Tumor Entities
NCT00351949	24	Completed	Stage IV Renal Cell Carcinoma	LAG-3	Biological: IMP321	IMP321 in Patients with Metastatic Renal Cell Carcinoma (MRCC)
NCT02676869	24	Completed	Stage IV Melanoma Stage III Melanoma	LAG-3 PD-1	Drug: IMP321 (eftilagimod alpha) Drug: Pembrolizumab	MP321 in Patients in Combination with Pembrolizumab in Patients with Unresectable or Metastatic Melanoma
NCT01968109	1499	Active, not recruiting	Neoplasms by Site	LAG-3 PD-1	Biological: Relatlimab Biological: Nivolumab Biological: BMS-986213	Anti-LAG-3 Monoclonal Antibody (BMS-986016) Administered Alone and in Combination with Anti-PD-1 Monoclonal Antibody (Nivolumab, BMS-936558) in Advanced Solid Tumors
NCT03044613	32	Active, not recruiting	Gastric Cancer Esophageal Cancer Gastroesophageal Cancer	LAG-3 PD-1	Drug: Nivolumab Drug: Relatlimab Drug: Carboplatin Drug: Paclitaxel Radiation: Radiation	Nivolumab or Nivolumab/Relatlimab Prior to Concurrent Chemoradiation in Patients with Operable Stage II/III Esophageal/ Gastroesophageal Junction Cancer
NCT04370704	144	Active, Recruiting	Melanoma	LAG-3 PD-1 TIM-3	Drug: INCAGN02385 Drug: INCAGN02390 Drug: INCMGA00012.	Combination Therapy with INCMGA00012 (Anti-PD-1), INCAGN02385 (Anti-LAG-3), and INCAGN02390 (Anti-TIM-3) in Participants with Select Advanced Malignancies

## Data Availability

Not applicable.
